# Examining the impact of learning about a resolved conflict on attitudes in an ongoing conflict: Evidence from the Israeli–Palestinian context

**DOI:** 10.1177/13684302251319689

**Published:** 2025-03-13

**Authors:** Deborah Shulman, Michal Reifen-Tagar, Noa Omri, Eran Halperin

**Affiliations:** 1 University of East Anglia, UK; 2 Reichman University, Israel; 3 Hebrew University of Jerusalem, Israel

**Keywords:** conciliatory policies, historical analogies, intergroup conflict, israeli–palestinian conflict, psychological interventions

## Abstract

A popular intervention for increasing support for peace in violent intergroup conflicts is to describe the peaceful resolution of other conflicts. In four experiments, we tested the effectiveness of this approach in the context of the Israeli–Palestinian conflict by exposing Jewish-Israelis to information about the Northern Ireland conflict and peace process, or about tourism in Northern Ireland as a control. We found that learning about the historical peace process generally led participants to view conflicts as more malleable, their own conflict as less unique, and led to unfreezing of conflict-related beliefs. However, it neither increased hope nor consistently boosted support for conciliatory policies. We explored boundary conditions and found effects were often stronger among leftist and centrist compared with rightists. Moreover, explicitly drawing analogies between conflicts at the outset proved ineffective, whereas exposing participants to the historical conflict and peace process without mentioning the proximal conflict was more successful.

In protracted violent intergroup conflicts, those involved often view peace as unattainable and oppose negotiating with the other side (Bar-Tal et al., 2010). Without public support for dialogue and concessions, spirals of conflict continue, often with catastrophic consequences. Activists, educators, and politicians frequently seek to boost support for conflict resolution by sharing information about different intractable conflicts that were resolved through negotiation. Such historical analogies are widespread in political discourse (Guelke, 2004). For example, during “the Troubles” in Northern Ireland, lessons were frequently drawn from the largely nonviolent struggles that brought an end to apartheid in South Africa to persuade parties in Northern Ireland to use nonviolent methods to achieve their political aims (Guelke, 1994). Those hoping to promote nonviolent conflict resolution in various ongoing conflicts have since attempted to draw lessons from the successful Northern Ireland peace process (Dudai, 2024; Mitchell, 2023; Senior, 2018). Although analogies are often employed strategically, it remains unclear whether, and how, they play a causal role in shaping the attitudes of lay people who are exposed to them. In this work, we conducted four experiments to investigate the effects of exposure to a different conflict and peace process on attitudes towards an ongoing conflict in which one’s ingroup is involved (hereafter referred to as the proximal conflict).

## What Is Learning by Historical Analogy?

We define learning by analogy as a change in beliefs, confidence about one’s beliefs, or the development of new beliefs following exposure to a source case that shares some similarities with the target case (Levy, 1994). The function of analogies is to derive a new solution, prediction, or idea by identifying similarities between the source and target, partially mapping similarities between them, and then extending this mapping by transferring knowledge from the source to the target (Gick & Holyoak, 1983). Analogies are considered powerful cognitive tools that can facilitate learning and problem solving (Duit, 1991). Compared with direct messages, analogies encourage more elaborate processing, which may enhance their persuasiveness (Petty & Cacioppo, 1986; Whaley, 1991).

Historical analogies are not formal or strict analogies (e.g., two is to four as three is to six), rather, they are heuristic, involving the transfer of ideas and insights (Tubi et al., 2022). Essentially, they entail an inference that if two events across different times share characteristics in one respect, they potentially have additional similarities (Khong, 2020). People draw lessons from the past continually to further their understanding and consider the best course of action in an uncertain situation, and have been doing so for centuries (Ghilani et al., 2017). For example, over 500 years ago, Machiavelli recommended diligently examining the past to foresee the future and “to apply those remedies which had been used by the ancients, or, not finding any of those used, to think of new ones from the similarity of events” (Machiavelli, 1517, as cited in Ghilani et al., 2017, p. 276). When all else is equal and the future is unknown, it may be sensible to choose the path that has proven successful in similar past situations (Leira, 2017). Historical analogies have been used in attempts to shape a range of attitudes across domains. For instance, analogies between HIV and Covid-19 were drawn to provide warnings and lessons for the latter (Catlin, 2021). Analogies have been used to guide approaches to climate change (Tubi et al., 2022), and experimental work found that exposure to an analogy between medical diseases and climate change strengthened the belief that climate change requires mitigation (Raimi et al., 2017).

## Analogical Learning in the Intergroup Context

Although learning about a resolved conflict may be an effective way to promote support for peace, research suggests that analogies can be rejected by skeptical audiences who emphasize the differences between cases (Schuman & Rieger, 1992). Indeed, all analogies break down somewhere, as no two cases are identical (Harrison & Treagust, 2006). Historical analogies rely on emphasizing similarities while downplaying differences (Gilovich, 1981; see also Kornprobst, 2007; Noon, 2004). However, this may be reversed in the context of conflict, with people emphasizing differences and downplaying similarities. Specifically, when people hold entrenched conflict-supporting views (Bar-Tal et al., 2014; Saguy & Reifen-Tagar, 2022), they may be particularly reluctant, or find it inappropriate, to recognize similarities and draw lessons from other conflicts to their own (Beatty, 2017; Kudish et al., 2015).

Nonetheless, a few studies to date provide encouraging evidence of analogical learning in the intergroup domain. For example, research has shown that after White participants in the US were prompted to consider their responsibility for violent hate crimes committed in the name of White people, they then expressed less collective blame towards Muslims for violent hate crimes committed in the name of Islam (Bruneau et al., 2018, 2020). Presumably, participants transferred the idea that individuals are not personally responsible for their group member’s actions from the source case to the target case. Another set of studies in the context of Israeli–Palestinian conflict found that Jewish-Israeli participants judged ingroup-collective punishment towards Palestinian civilians more critically after being exposed to a similar case of harmdoing in an unrelated context (Shulman et al., 2020). Furthermore, this effect was mediated by the endorsement of general principles against collective punishment, indicating an analogical process of retrieving information (the principle that collective punishment is wrong) from one case and then applying it to the other. This suggests that analogies do not only help people learn about the unfamiliar, but can also lead people to update their beliefs about the already familiar.

The only research that we know of that tested the effects of learning about a resolved conflict on attitudes about a proximal conflict is reported in a master’s thesis by Lustig (2003, as cited in Salomon, 2004). A total of 68 Israeli high school students were randomly assigned to participate (vs. not participate) in a program that included learning about the Northern Ireland conflict. They found mixed evidence: learning about a different conflict and peace process did not reduce outgroup prejudice, but it appeared to increase perspective taking of the outgroup in an essay-writing task. Crucially, whether learning about a different conflict and peace process can actually increase support for concrete conciliatory policies remains to be explored.

We investigated whether learning about a resolved conflict increases support for conciliatory policies via multiple distinct psychological mechanisms. Drawing on literature identifying key psychological barriers to conflict resolution (Bar-Tal & Halperin, 2011; Friend & Mahlotra, 2019; Hameiri et al., 2014), we focused on hope as a central affective mechanism; beliefs about conflict malleability and conflict uniqueness as key cognitive mechanisms; and unfreezing as a fundamental psychological process. Although there may be additional mechanisms at work, these variables were selected as they represent important pathways that can influence attitudes toward conflict resolution. In the context of violent conflicts, hope counteracts apathy (Hasler et al., 2023), malleability beliefs challenge mindsets that frame conflicts as inherently unchangeable (Cohen-Chen et al., 2023), perceiving one’s conflict as less unique addresses exceptionalism narratives that justify conflict continuation (Kudish et al., 2015), and unfreezing captures the psychological shift from rigidity to openness to alternative perspectives (Ben-Ezer et al., 2024). Although some of these variables may be interrelated (as elaborated in the General Discussion), here we examined these constructs as independent mechanisms to evaluate their unique contribution to increasing support for conciliatory policies. Below, we describe how each mechanism may be activated by exposure to a historical conflict and peace process.

First, we considered that learning about a resolved conflict could increase hope for peace. Although analogies are often regarded as a cognitive process, they can also involve the transferral of emotions from a source to a target (Thagard & Shelley, 2001). Hope has been defined as an emotion that is often associated with a pleasant feeling related to the appraisal of imagining a better future (Cohen-Chen et al., 2017). In conflicts that are characterized by repeated cycles of violence, hopelessness can become embedded in the public sentiment, reducing support for compromises (Hasler et al., 2023; Leshem, 2017). Learning about a peace process in another context may facilitate mental exploration of a different future for one’s own conflict, consequently increasing support for conciliatory attitudes.

Second, exposure to a resolved conflict could break down beliefs that stand as psychological barriers to conflict resolution. One such detrimental belief is that intergroup conflicts are inherently fixed and unchangeable (Bar-Tal, 2007). Simply learning about a conflict that has been resolved directly challenges this belief and may in turn increase peace-supporting views in the proximal context. Historical examples set a precedent—if a course of events happened once, they are in the realm of possibility. Indeed, experimental work has found that manipulating the belief that conflicts are malleable led to increased support for compromises (Cohen-Chen et al., 2014). Another barrier to supporting conciliatory policies is the perception that one’s own conflict is unique and incomparable with other violent intergroup conflicts (Kudish et al., 2015). This belief in the uniqueness of one’s conflict may serve to justify the view that one’s conflict is irresolvable, leading to the conclusion that supporting compromises would be futile at best. Exposure to information about a similar resolved conflict may lead people to consider their own conflict within a broader context. If similarities are acknowledged, people may begin to question the assumed intractability of their own conflict and be more open to considering conciliatory approaches.

Finally, exposure to a different conflict and its resolution may prompt a process of unfreezing, a crucial first step in attitudinal change (Lewin, 1997). Unfreezing typically begins when a person encounters a new idea that contradicts their existing beliefs, inducing psychological tension. To resolve this tension, individuals may start to reevaluate their own attitudes (Bar-Tal & Hameiri, 2020). Learning about a historical peace process may introduce information that is inconsistent with one’s existing narrative (e.g., that negotiations can be effective), which may instigate unfreezing and lead to the reevaluation of conflict-supporting beliefs. Although unfreezing does not guarantee immediate change, it opens the door to potential shifts in perspective, creating a space where people can consider alternative viewpoints.

## The Current Research

We conducted four experiments to test whether learning about a peace process that helped to resolve a different violent intergroup conflict can increase support for conciliatory policies in the context of one’s own group’s conflict, and explored the psychological processes through which it may lead to change. Specifically, we tested the effects of learning about the Northern Ireland conflict and peace process that led to the Good Friday Agreement on Jewish-Israelis’ support for conciliatory policies in the context of the Israeli–Palestinian conflict. The Israeli–Palestinian conflict dates back approximately a century, and despite rounds of peace talks and numerous peace proposals, there is no indication that it or the oppression of the Palestinian people is coming to an end. The conflict includes periods of high-intensity violence, exerting a high toll on both populations (especially the Palestinian population) and making despair prevalent. Popular support for a negotiated agreement, which is key to peaceful resolution, is only reducing among Israelis and Palestinians (The Israel Democracy Institute, 2022; Palestinian Center for Policy and Survey Research, 2022).

The Good Friday Agreement that brought an end to the 30-year-long violent conflict in Northern Ireland is often hailed as a model of conflict resolution (Hughes, 2015) and is frequently used to inspire peace (Dudai, 2024; White, 2013). Numerous academic articles, books (Guelke, 1994; Irwin, 2012; Rynhold & Cohen, 2003), and editorial pieces (Flescher, 2023; Savren, 2016) have been written, and even documentaries produced (Handelman Smith, 2012), comparing the Northern Ireland and Israeli–Palestinian conflicts and highlighting lessons that can be learned from the Northern Ireland peace process. Northern Irish leaders, ex-combatants, and activists have even travelled to Israel to inspire Israeli-Jews to strive for peace, and Israelis and Palestinians have travelled to Northern Ireland with the goal of learning more about the conflict and its resolution (Kelman, 2019; Senior, 2018). Even today, as the Israeli–Palestinian conflict has reached unprecedented scale following the October 7, 2023 Hamas-led massacre and the ensuing war that Israel waged in Gaza, analogies between the two conflicts continue to be drawn regularly to increase support for negotiations and conflict resolution among Israelis (see Damelin, 2023; Spiegel, 2024).

We acknowledge that support for conciliatory policies in the Israeli–Palestinian context may take many forms (Hakim et al., 2022), but we largely focused on compromises for a two-state solution, as polls suggest that of all viable solutions, this receives the greatest support among Jewish-Israelis (The Israel Democracy Institute, 2022) as well as Palestinians in Israel and in Gaza (although it should be noted that among Palestinians in the West Bank, a binational one-state solution receives greater support; Friedrich Ebert Stiftung, 2021).

We conducted four experiments, with students (Study 1) and the general public (Studies 2–4), and in times of relatively high- (Study 4), medium- (Studies 1 and 3), and low-intensity violence (Study 2) in Israel. On the one hand, during times of higher intensity violence, support for long-term peace may be overshadowed by immediate security concerns. On the other hand, as the urgency for conflict resolution may also be higher during such times, the intervention could be more effective (Pruitt, 2007; Zartman, 2000). Across our studies, we aimed to test the mediating effects of hope, conflict malleability beliefs, perceived uniqueness of the conflict, and unfreezing (Studies 3 and 4 only) on support for conciliatory policies. We also explored boundary conditions affecting the intervention’s effectiveness. In Study 3, we examined the impact of an analogy when explicitly drawn by an ingroup member at the outset. In Study 4, we aimed to isolate the effectiveness of explicitly presenting the analogy at the outset before learning about the other conflict, unlike in Study 3, where multiple factors were combined when the analogy was endorsed by an ingroup member. Additionally, we tested the moderating role of political ideology, and specifically, whether the interventions’ impact differed among leftists (known as “doves”), centrists, and rightists (known as “hawks”).

All supplemental materials and data from these studies are openly available on the Open Science Framework: https://osf.io/zjcg5/?view_only=03fd71a53e2d4bd3aa8b64161b3db6b3 (OSF).

## Study 1

In Study 1, we set out to test whether learning about the conflict and peace process in Northern Ireland would lead Jewish-Israelis to (a) have greater hope for peace, (b) believe conflicts in general are more malleable, (c) perceive the Israeli–Palestinian conflict as less unique, and (d) show greater support for conciliatory policies. Furthermore, we planned to test whether hope and beliefs would mediate the relationship between learning about the peace process in Northern Ireland and support for conciliatory policies. We conducted this study in May–June 2021 during and immediately following the 2021 Israel–Palestine crisis, which was a major outbreak of violence between Israel and Hamas as well as the Palestinian Islamic jihad in Gaza, which involved internal violence between Palestinian and Israeli-Jewish citizens of Israel. Due to the unforeseen escalation in violence, together with this being an exploratory study, Study 1 was not preregistered.

### Methods

#### Participants and procedure

A power analysis using G*Power 3.1 (Faul et al., 2009) found that obtaining a medium effect size (*d* = 0.50), with an alpha of .05 and power of 0.8, required 120 participants. We advertised the study for psychology students at two Israeli universities, and posted it on social media and kept it active until the end of the semester (approximately 3 weeks). A total of 221 participants completed the study. We screened out participants who failed any of the video-check or attention check questions, completed the survey more than once (checked based on university IDs), completed the study in a very short time (less than 400 s, which included the time watching the video), and those who did not identify as Jewish. After screening, 178 participants remained (age: *M* = 24.81, *SD* = 4.60; gender: 44 men and 134 women; political ideology: leftists = 57, centrists = 52, rightists = 69).

Participants were informed that they were taking part in a study about media and public opinion that involved watching a video clip and responding to several questions. Participants were randomly assigned to an experimental condition, hereafter called the peace analogy condition, or a control condition. In the peace analogy condition, participants watched a 3-min video clip about the Northern Ireland conflict and the Good Friday Agreement, with no mention of the Israeli–Palestinian conflict. In the control condition, participants watched a 3-min video clip about travelling in Belfast, with no mention of either conflict. Both were in English with Hebrew subtitles. Participants then responded to a survey in Hebrew. For compensation, participants who were psychology students received credits, while those who were recruited via social mediae were entered into a raffle for vouchers.

#### Measures

All responses to items across studies were on a 7-point scale (1 = *not at all*, 7 = *very much*) unless stated otherwise.

#### Mediators

##### Conflict malleability

Participants rated their agreement with three statements from Cohen-Chen et al. (2014): “Under certain circumstances and if all core issues are addressed, the nature of conflicts can be changed”; “The inherent characteristics of conflicts cannot be changed since their nature is fixed and unchanging” (reverse-coded item); and “Conflicts may seem at times like they are being resolved, but their true underlying nature will never change” (reverse-coded item). Higher scores indicate a stronger belief in conflict malleability.

##### Perceived uniqueness of the Israeli–Palestinian conflict

This was measured with five items adapted from Kudish et al. (2015): “The Israeli–Palestinian conflict is unique and cannot be compared to other conflicts”; “The Israeli–Palestinian conflict does not have special characteristics that make it unique” (reverse-coded item); “It is a mistake to think that Israelis and Palestinians can learn from other historical conflicts, because these conflicts are less complicated than the Israeli–Palestinian conflict”; “When I think about other conflicts in the world, I do not see a connection between them and the Israeli–Palestinian conflict”; and the fifth item asked “To what extent do you view the Israeli–Palestinian conflict and Northern Ireland conflict as different or similar?” with answers ranging from 1 (*very different*) to 7 (*very similar*) (reverse-coded item).^1^ High scores indicate a stronger belief that the Israeli–Palestinian conflict is unique.

##### Hope

To measure hope for peace, we included 10 items covering three dimensions: wishes for peace (three items), expectations for peace (three items), and the ability to imagine peace (four items). Wishes and expectations items were from Leshem (2017). Participants were asked if they either wished for or expected Israeli–Palestinian peace in the future, respectively, without detailing a particular solution to the conflict. The additional four items captured participants’ ability to imagine a different future. Participants were asked the extent to which they could imagine scenarios representing peace in the future (e.g., leaders of both sides shaking hands, friendships between children from both sides).

#### Outcome

##### Support for conciliatory policies

This was measured with items that are frequently used to measure support for compromises in the Israeli–Palestinian context (e.g., Alkoby et al., 2017; Halperin & Bar-Tal, 2011 Shulman et al., 2021), as they relate to core issues of the conflict and appeared in public discourse at the time of the study. Participants were asked the extent to which they supported Israel dividing Jerusalem and withdrawing to 1967 borders as part of a peace agreement. These two items focused on territorial compromises in exchange for peace. We also asked to what extent participants supported compensating Palestinian refugees, but the reliability of the measure was weaker with this item included, and therefore we omitted it.

#### Additional variables

In addition to demographics, our survey included variables to explore secondary questions. We measured familiarity with the Northern Ireland conflict to assess the novelty of the experimental information and its potential impact on the intervention’s effectiveness. We also examined behavioral intentions with two items: willingness to engage in a peaceful protest for peace, justice, and security alongside Palestinians; and willingness to donate to an Israeli–Palestinian peace NGO, to test if the interventions might motivate behaviors as well as change attitudes. However, we considered intentions would be difficult to shift as, according to the theory of planned behavior, they are shaped not only by attitudes but also by subjective norms and perceived behavioral control (Ajzen, 1991). In the context of the Israeli–Palestinian conflict, such behaviors are not normative (Pearlman, 2012) nor easy to enact (Wright, 2018). Finally, we measured attitudes towards Palestinians as a more distal outcome (items for all measures are in the Supplemental Material).

### Results and Discussion

Means, standard deviations, and correlations between main variables are presented in Table 1. We conducted a series of analyses of covariance (ANCOVAs) to test the effect of the intervention on our hypothesized mediators and outcomes, controlling for gender. We controlled for gender as the distribution of participants’ gender was significantly different between the two conditions, despite random assignment.

**Table 1.** Means, standard deviations, Cronbach’s alphas, number of items in measure, and correlation coefficients for key variables.

	**Study 1**							
Measure	*M*	*SD*	α (no. of items)	Pearson correlation				
				1	2	3	4	
1. Conflict malleability	4.75	1.14	.72 (3)	1				
2. Uniqueness of own conflict	4.09	1.19	.77 (5)	−.32***	1			
3. Hope	4.22	1.04	.83 (10)	.30***	−.27***	1		
4. Support for conciliatory policies	3.17	1.80	.79 (2)	.30***	−.32***	.37***	1	
	**Study 2**							
Measure	*M*	*SD*	α (no. of items)	Pearson correlation				
				1	2	3	4	
1. Conflict malleability	4.61	1.17	.73 (3)	1				
2. Uniqueness of own conflict	4.48	1.39	.84 (5)	−.36***	1			
3. Hope (ability to imagine peace)	3.84	1.55	.81 (4)	.37***	−.41***	1		
4. Support for conciliatory policies	4.45	1.68	.96 (10)	.39***	−.51***	.58***	1	
	**Study 3**							
Measure	*M*	*SD*	α (no. of items)	Pearson correlation				
				1	2	3	4	5
1. Conflict malleability	4.58	1.21	.74 (3)	1				
2. Uniqueness of own conflict	4.69	1.39	.84 (5)	−.43***	1			
3. Hope (ability to imagine peace)	3.49	1.52	.82 (4)	.39***	−.54***	1		
4. Unfreezing	2.53	1.72	N/A (1)	.26***	−.46***	.39***	1	
5. Support for conciliatory policies	4.32	1.73	.96 (10)	.35***	−.61***	.62***	.37***	
	**Study 4**							
Measure	*M*	*SD*	α (no. of items)	Pearson correlation				
				1	2	3	4	5
1. Conflict malleability	4.56	1.22	.78 (4)	1				
2. Uniqueness of own conflict	4.82	1.40	.84 (5)	−.50***	1			
3. Hope (wish for peace)	5.28	1.87	.88 (3)	.36***	−.33***	1		
4. Unfreezing	2.31	1.52	.90 (3)	.30***	−.37***	.22***	1	
5. Support for conciliatory policies	4.14	1.82	.97 (10)	.44***	−.60***	.55***	.38***	1
6. Support for agreement (single item)	4.14	2.18	N/A (1)	.38***	−.51***	.50***	.41***	.83***

*Note.* N/A = not applicable.

*** *p* < .001.

#### Conflict malleability beliefs

As expected, we found significant differences in malleability beliefs between conditions, *F*(1, 175) = 12.79, η^2^_p_ = .07, *p* < .001, with those in the peace analogy condition viewing conflicts as more malleable (*M* = 5.05, *SE* = 0.12, 95% CI [4.82, 5.28]) than those in the control condition (*M* = 4.45, *SE* = 0.12, 95% CI [4.22, 4.68]).

#### Perceived uniqueness of the Israeli–Palestinian conflict

There were significant differences between conditions in the extent to which the Israeli–Palestinian conflict was perceived as unique, *F*(1, 175) = 13.38, η^2^_p_ = .07, *p* < .001. Those in the peace analogy condition viewed the Israeli–Palestinian conflict as less unique (*M* = 3.77, *SE* = 0.12, 95% CI [3.53, 4.01]) compared with those in the control condition (*M* = 4.40, *SE* = 0.12, 95% CI [4.16, 4.64]).

#### Hope

We surprisingly found no significant effect of the intervention on hope for peace, *F*(1, 175) = 1.23, η^2^_p_ = .01, *p* = .269 (peace analogy condition: *M* = 4.30, *SE* = 0.11; control condition: *M* = 4.13, *SE* = 0.11).

#### Support for conciliatory policies

We found a significant difference between conditions in the expected direction, *F*(1, 175) = 4.48, η^2^_p_ = .03, *p* = .036. Participants in the peace analogy condition had significantly greater support for conciliatory policies (*M* = 3.46, *SE* = 0.19, 95% CI [3.09, 3.84]) than those in the control condition (*M* = 2.89, *SE* = 0.19, 95% CI [2.52, 3.26]).

#### The mediating role of conflict malleability beliefs and perceived uniqueness

**Figure 1.**
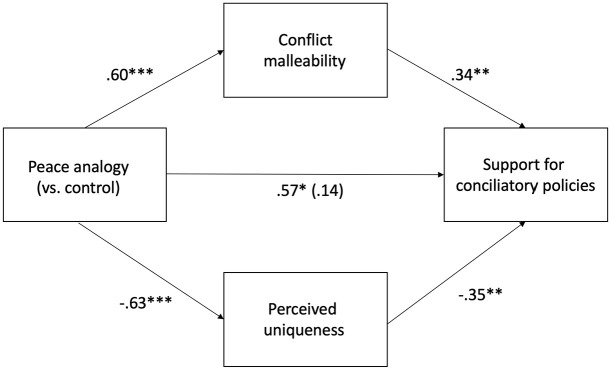
Conflict malleability beliefs and perceived uniqueness of the Israeli–Palestinian conflict as parallel mediators of the effect of the peace analogy (vs. control) condition on support for conciliatory policies, controlling for gender: Study 1. *p < .050. **p < .010. ***p < .001.

We examined the indirect effect of the peace analogy on increased support for conciliatory policies via changes in beliefs: believing that conflicts are inherently more malleable, and perceiving the Israeli–Palestinian conflict as less uniqueness. As the intervention did not impact hope, we did not include it as a mediator. A parallel mediation model using Hayes’s PROCESS macro (Model 4) revealed that the indirect effect of the peace analogy condition (vs. control) on support for compromises through malleability beliefs (effect = 0.21, *SE* = 0.09, 95% CI [0.05, 0.42]) and perceived uniqueness of the Israeli–Palestinian conflict (effect = 0.22, *SE* = 0.09, 95% CI [0.06, 0.41]) was significant, providing evidence in support of conflict malleability beliefs and perceived uniqueness of one’s own conflict as mediators (see Figure 1).

#### Additional findings

There were no effects of the intervention on attitudes towards Palestinians, suggesting that the effects of learning about a peace agreement in a different context do not spill over to attitudes towards the outgroup. This is consistent with Lustig’s (2003) findings that learning about the Northern Ireland conflict did not change levels of prejudice towards Palestinians (as cited in Salomon, 2004). Additionally, the intervention did not increase intentions to engage in peace activism. Although this could be considered a downstream consequence of supporting conciliatory policies, in the context of the Israeli–Palestinian conflict, very few people engage in peace activism alongside Palestinians and such actions meet large resistance from the public (Feinstein & Ben-Eliezer, 2019). We found no moderation by political ideology; however, it should be noted that our sample was underpowered for detecting small moderation effects. Finally, participants were generally unfamiliar with the conflict in Northern Ireland (the mean response for familiarity being 1.75 on a 1–7 scale; 1 = *unfamiliar*, 7 = *very familiar*). Familiarity with the conflict did not moderate the effect of the intervention.

Overall, the results of Study 1 suggested that learning about a peace agreement in a different context can increase support for conciliatory policies. Furthermore, we identified strengthened belief in the malleability of conflicts and weakened belief in the uniqueness of the proximal conflict as mechanisms for this effect. Our goal in Study 2 was to replicate our main findings. We also aimed to test whether the effect of the intervention was moderated by political ideology, and specifically, whether leftists, who hold more peace-supporting views (Leshem & Halperin, 2020; Pliskin et al., 2014), would be more affected by the intervention compared with rightists.

## Study 2

Study 2 was conducted among a sample of Jewish-Israelis from the general public recruited via a survey company. The study design was identical to that of Study 1, except for some changes to measures described below. This study was conducted in mid-February 2022, when levels of violence were relatively low.

We recruited a larger sample to test whether the effect of the intervention was moderated by political ideology. We considered that effects may be stronger for leftist compared with rightist participants. Existing research suggests that individuals with conservative/right-wing ideology are less open to new information that is incongruent with their attitudes (Jost et al., 2007). Moreover, work in the context of the Israeli–Palestinian conflict found that messages of hope changed leftists’, but not rightists’, attitudes about the conflict (Cohen-Chen et al., 2020). We also added quality checks, assessing whether participants found the videos interesting and informative. We increased the number of items in our outcome measure—support for conciliatory policies. This study was preregistered (https://aspredicted.org/4jcw-zf4s.pdf).

### Methods

#### Participants and procedure

We computed the required sample size assuming 80% power and a small interaction effect (Cohen’s *f* ^2^ of .02). This calculation suggested we required 395 participants. We aimed to recruit 425 to allow for screening out those who failed attention checks. The survey company recruited additional participants, and a total of 474 participants completed the main measures in the survey. In this study and the following ones, we aimed to recruit similar numbers of leftists and rightists to test moderation effects. Those who failed the video-check questions immediately following the manipulation were automatically directed out of the study and did not complete the survey. After screening out participants who failed either of two further attention check questions or completed the study in less than 400 seconds, consistent with our preregistration, 440 participants remained (age: *M* = 43.70, *SD* = 14.17; gender: 174 men, 264 women, one nonbinary, one missing; political ideology: leftists = 140, centrists = 142, rightists = 157, one missing).

#### Measures

We used the same items as in Study 1 to measure two mediators—conflict malleability beliefs and perceived uniqueness of the Israeli–Palestinian conflict.

#### Hope

Hope was measured with four items that captured the ability to imagine peace, which was previously part of our longer hope measure, to keep the length of the survey manageable.^2^

#### Support for conciliatory policies

In Study 2, we expanded our outcome measure to include a more comprehensive set of 10 items designed to capture a broader spectrum of attitudes toward conciliatory policies. Seven items focused on support for concessions, such as freezing the expansion of settlements, evacuating territories in the West Bank, and supporting the establishment of a Palestinian state. The remaining three items assessed support for the negotiation process, including endorsing dialogue between the Israeli and Palestinian leaderships. This expanded measure was developed to enhance the reliability and conceptual coverage of the construct.

In addition to testing the moderating role of political ideology, we also measured ingroup glorification (Leidner et al., 2010) to explore whether those who glorify the ingroup would be less affected by the intervention compared with those who do not. We measured ingroup attachment in order to control for this when testing the moderating effects of ingroup glorification (see Schori-Eyal et al., 2015).

## Results and Discussion

Means, standard deviations, and correlations between main variables are presented in Table 1. As preregistered, we ran analyses of variance (ANOVAs) to examine the effect of the peace analogy on our hypothesized mediators and support for conciliatory policies.^3^

### Conflict malleability beliefs

We found that there was a significant difference between conditions, *F*(1, 438) = 19.23, Cohen’s *d* = 0.41, *p* < .001, in the expected direction, such that those in the peace analogy condition (*M* = 4.86, *SD* = 1.23, 95% CI [4.69, 5.03]) viewed conflicts as more malleable than those in the control condition (*M* = 4.38, *SD* = 1.07, 95% CI [4.24, 4.52]).

### Perceived uniqueness of the Israeli–Palestinian conflict

Unlike in Study 1, there was no difference between conditions in perceived uniqueness of the Israeli–Palestinian conflict, *F*(1, 438) = 0.15, *p* = .701 (peace analogy: *M* = 4.51, *SD* = 1.48; control: *M* = 4.45, *SD* = 1.31).

### Support for conciliatory policies

Contrary to our hypothesis, we also found no significant difference between conditions in support for conciliatory policies, *F*(1, 438) = 0.22, *p* = .642 (peace analogy: *M* = 4.41 *SD* = 1.72; control: *M* = 4.48, *SD* = 1.65).

### Hope (the ability to imagine peace)

The difference in hope between conditions was in the expected direction, but not significant, *F*(1, 438) = 3.03, *p* = .083 (peace analogy: *M* = 3.98, *SD* = 1.55; control: *M* = 3.73, *SD* = 1.53).

We did not conduct mediation analyses as we did not find a main effect of condition on support for conciliatory policies.

### Moderation by political ideology

Political ideology moderated the effect of the peace analogy condition (vs. control) on malleability beliefs (*B* = −0.19, *SE* = 0.08, *t* = −2.45, *p* = .015). The intervention significantly strengthened the belief that conflicts are malleable among leftists (*B* = 0.76, *SE* = 0.15, *t* = 5.27, *p* < .001) and centrists (*B* = 0.51, *SE* = 0.10, *t* = 5.02, *p* < .001), but not rightists (*B* = 0.26, *SE* = 0.14, *t* = 1.82, *p* = .070). Similarly, ideology moderated the effect of the intervention on perceived uniqueness of the Israeli–Palestinian conflict (*B* = 0.19, *SE* = 0.09, *t* = 2.16, *p* = .032), indicating effects in the expected direction for leftists but not rightists. However, when examining the simple slopes for leftists, centrists, and rightists, none reached statistical significance. There was no moderation by ideology on hope nor on support for conciliatory policies. Furthermore, the effects of condition were not moderated by ingroup glorification, regardless of whether we controlled for ingroup attachment.

### Additional findings

Learning about the Northern Ireland conflict did not change intentions to engage in peace activism or outgroup attitudes, consistent with Study 1 findings. Our quality-control checks indicated that participants in the peace analogy condition found learning about the Northern Ireland conflict and Good Friday Agreement to be interesting (*M* = 4.86 on a scale of 1–7), and the material to be informative (4.90 on a scale of 1–7), and participants across conditions were quite unfamiliar with the Northern Ireland context (*M* = 2.76 on a 1–7 scale).

In Study 2, we found that although the peace analogy changed attitudes about conflicts in general, specifically leading participants to believe that conflicts are more malleable, it did not significantly change perceptions about the Israeli–Palestinian conflict’s uniqueness nor support for compromises. Participants found learning about the Northern Ireland conflict and Good Friday Agreement interesting and acquired new information, suggesting the null effects were not due to poor engagement with the material.

## Study 3

Given the mixed effects of learning about a different conflict and peace process on support for conciliatory policies, we explored ways to strengthen the effect of the intervention. Like in Study 1, this study was conducted during a period of active conflict. We ran the study at the beginning of May 2022, following a wave of Palestinian terrorist attacks in Israel that began in March, which were followed by Israeli military operations in the West Bank (Kingsley, 2022). This study was preregistered (https://aspredicted.org/h22b-hjdx.pdf).

In this study, we introduced a third condition designed to strengthen the effectiveness of the peace analogy. Drawing on self-categorization theory, we considered that participants would more likely apply lessons from the different conflict if the analogy were explicitly endorsed and presented by a prototypical ingroup member (Turner, 1991; for a review, see Spears, 2021). Ingroup members are usually deemed more trustworthy and credible (Woitzel et al., 2024). When people lack specific knowledge, they tend to assume that members of their ingroup, who share their social reality, can provide valuable information, making their messages more persuasive (McGarty et al., 1993; Platow et al., 2000). Indeed, research has found that attitude change appeals are often more effective when coming from ingroup members (e.g., MacKie et al., 1992; Wyer, 2010). Therefore, in this new condition, participants were first asked to read an article written by a prototypical ingroup member. The ingroup member shared reflections from a trip to Northern Ireland and mentioned lessons that could be learned and applied to the Israeli–Palestinian conflict. The text was based on existing online material but adapted for research purposes. After reading the article, participants watched the intervention video about the conflict in Northern Ireland and peace process. We expected the ingroup-endorsed peace analogy to be the most effective.

Furthermore, we tested whether being exposed to information about a different conflict and peace process unfreezes beliefs about the Israeli–Palestinian conflict, and whether this may explain increased conciliatory attitudes. Previous work has found that psychological interventions can initiate processes of unfreezing, increasing openness to contradictory views and increasing support for peace (Bar-Tal & Hameiri, 2020; Hameiri et al., 2014; Wohl et al., 2016). The rationale for using analogies to increase support for conciliatory policies was that they provide a new lens through which to view conflicts, potentially destabilizing fixed beliefs and attitudes. However, until this study, we had not tested unfreezing as a mechanism.

### Methods

#### Participants and procedure

We recruited a sample of Jewish-Israelis from the general public through an online survey company. Given the inconsistent results of the previous studies, we wanted to ensure that we had a large enough sample to detect small to medium effects. We found that obtaining such an effect size (*d* = 0.35), with an alpha of .05 and power of 0.8 would require at least 130 participants per condition. We aimed to collect data from 430 participants to allow for screening, but extra participants were recruited by the survey company. Those who failed the video-check questions were automatically directed out of the study. After screening out participants who failed any further attention check questions or completed the study in less than 400 s, in line with our preregistered screening criteria, 473 participants remained (age: *M* = 42.33, *SD* = 13.56; gender: 222 men, 248 women, three missing; political ideology: leftists = 142, centrists = 120, rightists = 208, three missing).

Participants were randomly assigned to one of three conditions: control, original peace analogy, and ingroup-endorsed peace analogy. As in the previous studies, participants in the original peace analogy condition watched the video about the Northern Ireland conflict and peace process, and those in the control condition watched the video about tourism. In the ingroup-endorsed peace analogy condition, participants first read a fabricated article in the style of a blog written by a prototypical ingroup member (a Modern- Orthodox Jewish rabbi) that drew a direct analogy between the conflicts and highlighted lessons that could be applied to the Israeli–Palestinian conflict (full article is in the Supplemental Material), before watching the video about the Northern Ireland conflict and peace process.

#### Measures

We used identical items to those in Study 2 to measure conflict malleability beliefs, perception of the Israeli–Palestinian conflict as unique, hope (ability to imagine peace), and support for conciliatory policies. We added a one-item measure of unfreezing at the end of the survey based on a one-item measure used by Hameiri et al. (2018): “To what extent did the blog post/video that you watched lead you to reevaluate your beliefs about the Israeli–Palestinian conflict?” with a 7-point response scale (1 = *not at all*, 7 = *to a great extent*). As in the previous studies, we measured outgroup attitudes and intentions to engage in peace activism. We also asked if the information in the video was interesting and informative as in the previous studies, we also measured reactance (Dillard & Shen, 2005) to assess whether participants felt manipulated by the analogy.

### Results and Discussion

Means, standard deviations, and correlations between main variables are presented in Table 1. We conducted ANOVAs with follow-up two-tailed tests to compare conditions.

#### Conflict malleability beliefs

We found a significant difference in malleability beliefs between conditions, *F*(2, 469) = 3.85, *p* = .022. Compared with those in the control condition (*M* = 4.41, *SD* = 1.17, 95% CI [4.23, 4.58]), those in the original peace analogy condition (*M* = 4.77, *SD* = 1.18, 95% CI [4.59, 4.95]) believed that conflicts were more malleable (*p* = .006), but the difference between those in the control condition and the ingroup-endorsed peace analogy condition (*M* = 4.58, *SD* = 1.27, 95% CI [4.36, 4.81]) was not significant (*p* = .212). There was also no significant difference between the original peace analogy and ingroup-endorsed peace analogy conditions (*p* = .193), altogether suggesting that the ingroup-endorsed peace analogy condition was in fact not more effective.

#### Perceived uniqueness of the Israeli–Palestinian conflict

There were no significant differences in uniqueness perceptions between conditions, *F*(2, 468) = 1.41, *p* = .246. A pairwise test also revealed no significant difference between any two conditions (control: *M* = 4.82, *SD* = 1.29; original peace analogy: *M* = 4.66, *SD* = 1.46; ingroup-endorsed peace analogy: *M* = 4.56, *SD* = 1.44).

#### Hope (ability to imagine peace)

The overall ANOVA showed that there was no main effect of condition, *F*(2, 470) = 2.09, *p* = .126. However, despite the nonsignificant main effect, pairwise comparisons may still uncover meaningful differences between specific conditions, providing a more detailed understanding of the effects of each condition that may be obscured in the overall ANOVA (Chen et al., 2018). Indeed, those in the original peace analogy condition (*M* = 3.62, *SD* = 1.58, 95% CI [3.38, 3.86]) were able to imagine peace significantly more than those in the ingroup-endorsed peace analogy condition (*M* = 3.26, *SD* = 1.51, 95% CI [3.00, 3.53]), *p* = .047). There was no significant difference between the control condition (*M* = 3.53, *SD* = 1.45, 95% CI [3.32, 3.75]) and either the peace analogy (*p* = .606) or the ingroup-endorsed peace analogy condition (*p* = .128).

#### Unfreezing

There was a significant difference between conditions, *F*(2, 468) = 20.60, *p* < .001. There were significant differences between the control condition (*M* = 1.89, *SD* = 1.42, 95% CI [1.68, 2.10]) and the original peace analogy condition (*M* = 2.89, *SD* = 1.76, 95% CI [2.62, 3.15], *p* < .001), and between the control condition and the ingroup-endorsed peace analogy condition (*M* = 2.92, *SD* = 1.80, 95% CI [2.60, 3.24], *p* < .001), such that those who were exposed to information about the Northern Ireland conflict and peace process reported reevaluating their beliefs about the Israeli–Palestinian conflict more than those in the control condition. There was no significant difference between the two analogy conditions (*p* = .868).

#### Support for conciliatory policies

We found a significant difference in support for conciliatory policies between conditions, *F*(2, 470) = 3.21, *p* = .041. Compared with the control condition (*M* = 4.14, *SD* = 1.72, 95% CI [3.89, 4.40]), those in the original peace analogy condition (*M* = 4.58, *SD* = 1.69, 95% CI [4.33, 4.84]) showed greater support for conciliatory policies (*p* = .017). However, the ingroup-endorsed peace analogy condition (*M* = 4.21, *SD* = 1.78, 95% CI [3.90, 4.52]) did not differ from the control condition (*p* = .743). The difference between the ingroup-endorsed peace analogy condition and the original analogy condition was not in the expected direction, with lower support for conciliatory policies for those in the ingroup-endorsed condition; however, this difference did not reach the threshold for significance (*p* = .063).

#### The mediating role of conflict malleability beliefs and unfreezing

**Figure 2.**
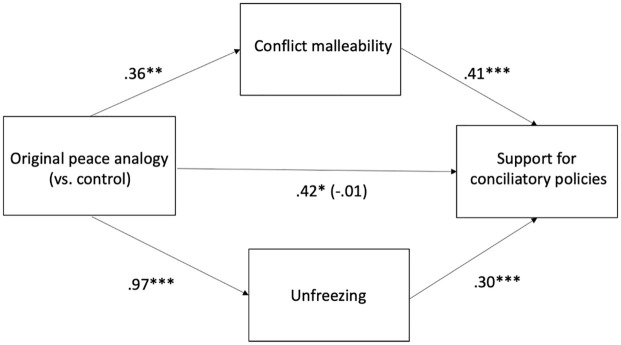
Conflict malleability beliefs and unfreezing as parallel mediators of the effect of the peace analogy (vs. control) condition on support for conciliatory policies: Study 3. *p < .05. **p < .01. ***p < .001.

We next tested a parallel mediation model in which a stronger belief that conflicts are malleable and unfreezing mediated the effect of the original peace analogy (vs. control) on support for conciliatory policies, using Hayes’s PROCESS macro (Model 4). Mediation analysis revealed that the indirect effect of the original peace analogy condition (vs. control) on support for conciliatory policies through conflict malleability beliefs (effect = 0.15, *SE* = 0.06, 95% CI [0.04, 0.27]) and unfreezing (effect = 0.29, *SE* = 0.08, 95% CI [0.16, 0.46]) was significant, providing support for conflict malleability beliefs and unfreezing as distinct mechanisms of the effect of the peace analogy on support for conciliatory policies (see Figure 2).

#### Moderation by political ideology

To test if ideology moderated the effects of the peace analogy interventions on the mediators and outcome, we performed multicategorical moderation analysis using Hayes’s (2018) PROCESS macro (Model 1). We used the indicator approach for multicategorical predictors, and the control condition was the reference group. PROCESS created two dummy variables: D1 reflected the control versus original peace analogy, and D2 reflected the control versus ingroup-endorsed peace analogy. For simple slope analysis, left-wing ideology was fixed at 1 *SD* below the mean, centrist ideology was the mean, and right-wing ideology was fixed at 1 *SD* above the mean. Significant moderation effects are presented in Table 2.

**Table 2.** The moderating effect of political ideology on the relationship between peace analogies (vs. control) and conflict-related beliefs: Study 3.

	Estimate	*SE*	*t*	*p*
**Conflict malleability beliefs**				
**D1 × Political Ideology interaction** Simple slopes	**−0.28**	**0.09**	−**3.02**	**.003**
Left	0.67	0.17	3.88	< .001
Center	0.29	0.12	2.34	.020
Right	−0.09	0.18	−0.51	.609
**Perceived uniqueness of the Israeli–Palestinian conflict**				
**D1 × Political Ideology interaction** Simple slopes	**0.26**	**0.10**	**2.67**	**.008**
Left	−0.41	0.18	−2.27	.024
Center	−0.06	0.13	−0.44	.662
Right	0.29	0.19	1.57	.116
**Unfreezing**				
**D1 × Political Ideology interaction** Simple slopes	**−0.29**	**0.13**	−**2.27**	**.024**
Left	1.34	0.24	5.60	< .001
Center	0.94	0.17	5.52	< .001
Right	0.55	0.25	2.19	.029
**D2 × Political Ideology interaction** Simple slopes	**−0.27**	**0.13**	−**2.01**	**.045**
Left	1.47	0.27	5.49	< .001
Center	1.10	0.19	5.96	< .001
Right	0.74	0.25	2.95	.003

*Note.* For each conflict-related belief measure (displayed in bold text), signficant interaction effects are followed by simple slopes analyses showing the effect of peace analogies at different levels of political ideology (left-wing = -1 SD, center = mean, right-wing = +1 SD). Effects for D1 compare the standard peace analogy condition to the control condition, and effects for D2 compare the ingroup-endorsed peace analogy condition to the control condition.

There was a significant moderation effect for the D1 (but not D2) comparison on conflict malleability beliefs. Leftists and centrists in the original peace analogy condition (vs. control condition) believed conflicts were more malleable, but rightists did not. Although there was no main effect on perceived uniqueness of the Israeli–Palestinian conflict, there was a moderated effect for the D1 comparison. In the original peace analogy condition (vs. control), leftists saw the Israeli–Palestinian conflict as less unique, but centrists and rightists did not. For unfreezing, there was a significant moderation effect for the D1 and D2 comparisons, with the effects of the intervention being largest for leftists, and increasingly smaller for centrists and then rightists. There was no moderation by political ideology on hope or support for conciliatory policies.

#### Additional findings

Those in the experimental conditions found learning about the Northern Ireland conflict and peace process informative (*M* = 5.44 on a 1–7 scale) and interesting (*M* = 5.22 on a 1–7 scale). The intervention did not induce substantial reactance (*M* = 2.06 on a 1–7 scale), suggesting that it did not make participants feel angry or manipulated. There were no significant differences between conditions in outgroup attitudes or behavioral intentions.

The comparison between the original analogy condition and the control condition largely replicated the results of Study 1. We found that watching a video about the Northern Ireland conflict and the peace process significantly increased Jewish-Israelis’ support for conciliatory policies. This effect was mediated by believing that conflicts are more malleable and greater unfreezing of beliefs about the Israeli–Palestinian conflict. There was no main effect of the original peace analogy condition (vs. control) on perceived uniqueness of the conflict, but the intervention led leftists and centrists, but not rightists, to view the Israeli–Palestinian conflict as less unique. The moderation effect did not extend to hope or conciliatory attitudes, altogether suggesting that the effects of peace analogy interventions are often, but not always, stronger for leftists and centrists, compared with rightists.

Contrary to our hypothesis, the ingroup-endorsed peace analogy did not enhance the effectiveness of the intervention and mainly had the same effect as the neutral control condition. Furthermore, participants in the original peace analogy condition reported greater ability to imagine peace than those in the ingroup-endorsed peace analogy condition. We consider that these unexpected findings may stem from the directness of the presentation of the analogy in the ingroup-endorsed analogy condition. The comparison between the Northern Ireland conflict and the Israeli–Palestinian conflict was drawn explicitly at the outset. In contrast, those in the original peace analogy condition were first presented with the Northern Ireland case independently, with the Israeli–Palestinian conflict only mentioned later in the survey questions. Research suggests that explicit analogies that mention the source and target cases simultaneously can impose more working memory load on the learner, as they require the learner to split their attention between cases, potentially hindering learning (Sidney & Thompson, 2019). Introducing the source case first, however, may facilitate deeper processing of new information (Gray & Holyoak, 2021). In our study, the explicitness of the analogy at the outset in the ingroup-endorsed condition may have inadvertently led to a more superficial, subjective, or ingroup-focused viewpoint when considering the Northern Ireland case, possibly impeding analogical transfer. In our next study, we aim to explore this possibility.

## Study 4

Study 4 aimed to isolate and test the effectiveness of a peace analogy presented explicitly at the outset, in contrast to Study 3, which combined multiple factors in its ingroup-endorsed peace analogy condition. In our previous study, we found that when an analogy was explicitly drawn by an ingroup member, it was largely ineffective. However, the simultaneous manipulation of factors in this condition (i.e., the analogy was endorsed by an ingroup member, the proximal conflict was mentioned at the same time as learning about the Northern Ireland conflict) made it difficult to identify the cause of its ineffectiveness. To address this, we teased these elements apart and introduced a condition where participants were simply informed that parallels are commonly drawn between the Northern Ireland and Israeli–Palestinian conflicts before receiving information about the Northern Ireland’s conflict and peace process. This allowed us to examine the specific role of explicitly drawing the analogy at the outset, eliminating potential confounding effects. We ran the study at the beginning of August 2024, during a time of unprecedented high-intensity conflict that began on October 7, 2023. This marked the deadliest attack on Israel in the country’s history, which led to an Israel’s military campaign (Israel–Hamas war), causing mass destruction in Gaza and tens of thousands of Palestinian deaths (Frankel, 2024). This study was preregistered (https://aspredicted.org/cknv-jrhb.pdf).

### Methods

#### Participants and procedure

We aimed to recruit a sample of 430 Jewish-Israelis from the general public through an online survey company. After screening out participants who failed attention check questions or completed the study in less than 400 s, as specified in our preregistration, 408 participants remained (age: *M* = 44.19, *SD* = 15.80; gender: 126 men, 282 women; political ideology: leftists = 124, centrists = 124, rightists = 160).

Participants were randomly assigned to one of three conditions: the original peace analogy condition, the control condition, both of which were identical to those in the previous studies, or the new explicit peace analogy condition. In this condition, participants were presented with the following text before watching the intervention video:

You will now watch a short video about the Northern Ireland conflict, which has often been compared to the Israeli–Palestinian conflict because of perceived similarities. This video provides an overview of the Northern Ireland conflict, known as the Troubles, and the peace process that ultimately led to the landmark Good Friday Agreement. Some political analysts have suggested Israelis and Palestinians could potentially draw insights from studying how Northern Ireland transitioned from violence to a negotiated political settlement.

In all other respects, the two peace analogy conditions were identical.

#### Measures

We used the same items as in the previous studies to measure conflict malleability beliefs, perception of the Israeli–Palestinian conflict as unique, and support for conciliatory policies. However, given the unprecedented level of conflict at the time of the study, we considered that support for conciliatory policies would likely remain unchanged, so we also added a separate single-item outcome that we considered might be more susceptible to change: support for a political–security arrangement. This item has been used in public opinion polls since October 7, 2023 (Halperin, 2024). Participants were asked to indicate their agreement with the following statement on a 7-point scale (1 = *not at all*, 7 = *very much*): “A political–security arrangement should be promoted under the leadership of the USA and with the support of the Arab countries, which would include security guarantees for Israel, and the establishment of a demilitarized Palestinian state.” In this study, we measured wishes for peace to capture hope. We also added two items to our measure of cognitive unfreezing to increase the reliability of this measure (see Supplemental Material). We included additional measures from the previous studies, including intentions to engage in peace activism and outgroup attitudes.

### Results and Discussion

Means, standard deviations, and correlations between main variables are presented in Table 1. We conducted ANOVAs followed by pairwise t tests, with each mechanism and outcome as dependent variables.

#### Conflict malleability beliefs

The overall ANOVA was significant, *F*(2, 405) = 7.38, *p* = .001. As in the previous studies, compared with those in the control condition (*M* = 4.29, *SD* = 1.21, 95% CI [4.10, 4.49]), those in the original peace analogy condition (*M* = 4.85, *SD* = 1.19, 95% CI [4.64, 5.06]) believed that conflicts were more malleable (*p* < .001). The mean of the explicit peace analogy condition (*M* = 4.57, *SD* = 1.19, 95% CI [4.36, 4.77]) fell between these two conditions, but did not differ significantly from either the control (*p* = .059) or the original peace analogy condition (*p* = .056).

#### Perceived uniqueness of the Israeli–Palestinian conflict

The overall ANOVA showed no main effect of condition, *F*(2, 405) = 2.21, *p* = .111. However, pairwise t tests showed a significant difference between the control (*M* = 4.98, *SD* = 1.25, 95% CI [4.78, 5.19]) and the original peace analogy condition (*M* = 4.63, *SD* = 1.51, 95% CI [4.37, 4.89], *p* = .036), indicating that the original analogy led people to view the proximal conflict as less unique, compared with the control. On the other hand, there were no significant differences between the control and the explicit analogy condition (*M* = 4.83, *SD* = 1.43, 95% CI [4.58, 5.07], *p* = .354), nor between the two analogy conditions (*p* = .248).

#### Hope (wishes for peace)

There was no significant difference in hope (wishes) for peace between conditions, *F*(2, 405) = 0.45, *p* = .636, nor between any two conditions (control: *M* = 5.16, *SD* = 1.99; original peace analogy: *M* = 5.29, *SD* = 1.93; explicit peace analogy: *M* = 5.38, *SD* = 1.66).

#### Unfreezing

There was a significant difference in unfreezing between conditions, *F*(2, 405) = 29.36, *p* < .001. Differences were significant between the control (*M* = 1.58, *SD* = 0.98, 95% CI [1.42, 1.75]) and the original peace analogy condition (*M* = 2.71, *SD* = 1.74, 95% CI [2.41, 3.01], *p* < .001), and between the control and the explicit peace analogy condition (*M* = 2.71, *SD* = 1.49, 95% CI [2.45, 2.97], *p* < .001), such that those who were exposed to the analogy reported reevaluating their beliefs about the Israeli–Palestinian conflict more than those in the control condition. There was no significant difference between the two peace analogy conditions (*p* = .994).

#### Support for conciliatory policies

We assessed support for conciliatory policies using two measures: a new single-item measure of support for a political–security arrangement, and our previous 10-item measure. For the single-item measure, the overall ANOVA was not significant, and we found no significant differences in support for conciliatory policies between conditions, *F*(2, 405) = 1.66, *p* = .191 (control: *M* = 3.88, *SD* = 2.20; original peace analogy: *M* = 4.32, *SD* = 2.22; explicit peace analogy: *M* = 4.26, *SD* = 2.11). The difference between the control and original peace analogy condition was in the expected direction, but did not reach the threshold for significance (*p* = .099). There were no differences in support for conciliatory policies between conditions using the previous 10-item measure, *F*(2, 405) = 0.49, *p* = .613 (control: *M* = 4.02, *SD* = 1.85; original peace analogy: *M* = 4.22, *SD* = 1.82; explicit peace analogy: *M* = 4.19, *SD* = 1.80). As we did not find a main effect of condition on conciliatory policies, we did not conduct mediation analyses.

#### Moderation by political ideology

To test if political ideology moderated the effectiveness of the interventions on the mediators and outcome, we performed multicategorical moderation analysis using Hayes’s (2018) PROCESS macro (Model 1), as in Study 3. PROCESS created two dummy variables: D1 reflected the control versus original peace analogy, and D2 reflected the control versus explicit peace analogy. Significant moderation effects are presented in Table 3.

**Table 3.** The moderating effect of political ideology on the relationship between each peace analogy condition (vs. control) and conflict-related beliefs: Study 4.

	Estimate	*SE*	*t*	*p*
**Perceived uniqueness of the Israeli–Palestinian conflict**				
**D1 × Political Ideology interaction**	**0.20**	**0.10**	**2.00**	**.046**
Left (simple slope)	−0.56	0.19	−2.90	.004
Center (simple slope)	−0.28	0.14	−2.05	.041
Right (simple slope)	−0.01	0.20	−0.03	.973
**Unfreezing**				
**D1 × Political Ideology interaction**	**−0.31**	**0.12**	**−2.70**	**.007**
Left (simple slope)	1.52	0.23	6.69	< .001
Center (simple slope)	1.08	0.16	6.64	< .001
Right (simple slope)	0.64	0.23	2.76	.006
**D2 × Political Ideology interaction**	**−0.24**	**0.12**	**−2.05**	**.041**
Left (simple slope)	1.48	0.23	6.35	< .001
Center (simple slope)	1.14	0.16	7.09	< .001
Right (simple slope)	0.81	0.23	3.61	< .001

*Note.* For each conflict-related belief measure (shown in bold text), signficant interaction effects are followed by simple slopes analyses showing the effect of peace analogies at different levels of political ideology (left-wing = -1 SD, center = mean, right-wing = +1 SD). Effects for D1 compare the standard peace analogy condition to the control condition, and effects for D2 compare the ingroup-endorsed peace analogy condition to the control condition.

Political ideology did not moderate the effect of condition on conflict malleability beliefs. There was a significant moderation effect on the perceived uniqueness of the Israeli–Palestinian conflict for the D1 comparison. In the original analogy condition (vs. control), leftists and centrists saw the Israeli–Palestinian conflict as less unique, but rightists did not. For unfreezing, there was a significant moderation effect for the D1 and D2 comparisons, with effects largest for leftists, then centrists, then rightists, but still significant for all groups. There was no moderation by political ideology on support for conciliatory policies. This pattern of moderation effects largely replicated that in Study 3.

#### Additional findings

Those in the experimental conditions found learning about the Northern Ireland conflict and peace process informative (*M* = 5.12 on a 1–7 scale) and interesting (*M* = 4.95 on a 1–7 scale). There were no significant differences in outgroup attitudes or behavioral intentions between conditions.

### Discussion

In Study 4, we found that the original peace analogy condition led participants to view conflicts as more malleable, view their own conflict as less unique, and reevaluate their beliefs about the Israeli–Palestinian conflict more (i.e., unfreezing), compared with the control condition. The explicit peace analogy condition did not lead participants to view conflicts as significantly more malleable nor did it change perceptions about the proximal conflict’s uniqueness. This suggests that drawing a direct comparison at the outset when using analogies to try to persuade is generally an ineffective approach, and likely explains the null results of the ingroup-endorsed analogy condition in Study 3. The effect of the analogy on perceived uniqueness of the Israeli–Palestinian conflict was moderated by political ideology, with effects present among leftists and centrists but not rightists, and unfreezing effects were also larger for leftists. Exposure to a peace analogy, explicit or implicit, did not increase support for conciliatory policies, compared with the neutral control condition. However, we did consider that it might be especially difficult to increase support for compromises given the unprecedent levels of conflict at the time the study was conducted.

## General Discussion

The current work examined whether exposure to an example of successful conflict resolution in an unrelated historical context can increase support for conciliatory policies in an ongoing proximal conflict. Although this strategy is commonly used by advocates for negotiated agreements, surprisingly little research has explored the psychological effects of peace analogies on those who encounter them. We therefore conducted four experimental studies to test this. Studies 1 and 3 demonstrated that learning about the Northern Ireland conflict and peace process increased Israeli-Jews’ support for conciliatory policies. This effect was mediated by believing conflicts are more malleable, perceiving the proximal conflict as less unique (Study 1 only), and reevaluating beliefs about the conflict (“unfreezing,” Study 3 only). In Studies 2 and 4, although we found no effect of peace analogies on support for conciliatory policies, they still shifted beliefs about conflict resolution. Thus, although the effects of drawing on a different conflict and peace process on increasing support for conciliatory policies were not consistently robust, they frequently resulted in changes in beliefs, indicating their potential to induce psychological change.

We tested whether *explicitly* drawing parallels between the Northern Ireland conflict and the Israeli–Palestinian conflict would further facilitate attitude change in Studies 3 and 4. Surprisingly, this explicit approach was largely ineffective at changing conflict-related beliefs, whereas simply presenting participants with information about the Northern Ireland conflict was often effective. This may be due to several factors. First, a direct approach may raise defensiveness. Indeed, it has been argued that when political–societal conditions are unfavorable to the development of peace education, an indirect approach may be more appropriate (Bar-Tal & Rosen, 2009). An indirect approach teaches topics such as reflective thinking and conflict resolution skills, but avoids delving into issues about the specific conflict, its history, and the rival group. Second, an explicit approach bypasses the potentially valuable process of participants generating their own insights. Research on self-generated knowledge suggests that when individuals independently discover connections and actively draw their own conclusions, they engage in deeper processing and show stronger attitude change (Chi et al., 1994). Third, the specific analogy may have been perceived as relatively weak. Despite surface similarities in religious–political divisions and territorial disputes, key differences exist between the conflicts. Israeli commentators have often argued that the Israeli–Palestinian conflict involves fundamentally different stakes and dynamics, such as questions of state legitimacy and survival that were not present in the Northern Ireland case (see Dudai, 2024). Making these comparisons explicit may have heightened awareness of perceived differences, potentially undermining the intervention’s effectiveness.

We found that political ideology moderated several of the effects of the analogies in Studies 2, 3, and 4, where we had larger sample sizes to better detect such effects. Specifically, the effects of the analogy on the hypothesized psychological processes—conflict malleability beliefs, perceptions of the Israeli–Palestinian conflict as unique, and unfreezing—were often larger or only present for leftists and sometimes centrists in the analogy (vs. control) condition, but generally not for rightists. This is consistent with findings from other studies that suggest leftists may be more receptive to messages designed to increase conciliatory attitudes in the context of the Israeli–Palestinian conflict (e.g., Cohen-Chen et al., 2020). Although this peace intervention was less effective among those with a more hawkish ideology, our findings suggest it did not merely preconfirm existing beliefs among dovish audiences, as it led to measurable attitude change. Moreover, the observed shifts among centrists indicate that this intervention may have broader potential impact.

We conducted this series of studies across varying levels of conflict intensity: relatively low (Study 2), moderate (Studies 1 and 3), and high (Study 4). We found that the intervention increased support for conciliatory policies during periods of moderate conflict intensity only (Studies 1 and 3). This pattern suggests that the intervention’s effectiveness may follow a curvilinear relationship with conflict intensity. This hypothesis would align with readiness (Pruitt, 2007, 2015) and ripeness theories (Zartman, 2000, 2022), which both propose that optimal conditions for conflict resolution emerge when a conflict is sufficiently salient but not too intense as to extinguish any desire for peace. Specifically, readiness theory proposes that both motivation to de-escalate and optimism about reaching a mutually acceptable agreement are prerequisites for a reconciliatory approach. In low-intensity periods of a conflict, motivation may be insufficient as the costs inflicted by conflict are low. This lack of motivation may make people less receptive to learning from other conflicts and peace processes, as they perceive little urgency in addressing the proximal conflict. Conversely, during times of extremely intense conflict, while motivation might be high due to conflict costs, optimism—the belief that negotiations can lead to an acceptable agreement—may be severely compromised, and support for aggressive policies may dominate (see Shani et al., 2024). During these times, people might find it particularly difficult to draw lessons from other peace processes, as they may view their current conflict as irresolvable via negotiations. At moderate levels of conflict intensity, however, both motivation and optimism may be present at sufficient levels simultaneously, creating optimal conditions for engaging with, and drawing lessons from, the source case. Further research is needed to systematically test this hypothesized relationship between conflict intensity and the intervention’s effectiveness, including examining the psychological mechanisms that may explain this potential curvilinear pattern.

### Limitations and Future Directions

This work has several limitations that suggest avenues for future research. First, we tested the effect of learning about the resolution of a specific conflict—the Northern Ireland conflict—on a single group: Jewish-Israelis. We selected this analogy due to how frequently it is used to instil lessons (Dudai, 2024). Although this provides valuable insights into how analogies operate in the context of acute, violent conflict, it is unclear whether effects will generalize to Palestinians, or to other conflict contexts. This is particularly important given the power asymmetries that are common in intergroup conflicts, including in the Israeli–Palestinian context (Ross, 2014; Rouhana, 2018). Drawing on the needs-based model of reconciliation (Shnabel & Nadler, 2008), which posits that victim and perpetrator groups have distinct psychological needs that must be addressed for reconciliation to occur, future research could explore whether victim and perpetrator groups require different types of analogies to resonate with their experiences and goals. For instance, groups experiencing systematic oppression and denial of basic rights may find analogies focusing on historically oppressed groups achieving empowerment, justice, and political change (e.g., the anti-apartheid movement in South Africa) more impactful. These narratives, which highlight overcoming radically asymmetric power relations, may resonate more than those emphasizing compromise and cooperation.

Second, we relied on self-report measures to evaluate the intervention. Although this is very common practice when assessing psychological interventions in this field (e.g., Čehajić-Clancy et al., 2011; Hasson et al., 2019), such measures may not always accurately capture attitudes (Bertrand & Mullainathan, 2001). To explore other indicators of change, future research could use brain imaging techniques, such as functional magnetic resonance imaging (fMRI) or magnetoencephalography (MEG), to record brain activation associated with problem solving or effective persuasion (see Levy et al., 2024), or assess actual behavioral change, such as voting patterns following the intervention (see Hameiri et al., 2014). Despite this limitation, we believe that in the context of intergroup conflict, self-reported attitudes remain valuable as they can influence voting and policy change (Halperin et al., 2014).

Third, in the control condition, participants watched a video about tourism in Northern Ireland, which was matched to the experimental video in both the location featured and duration. The control video may have not sufficiently matched the emotional engagement of the experimental video. For example, the experiment video featured dramatic music when describing the conflict, followed by uplifting music when describing the peace process. Heightened emotions could facilitate the effect of learning (Forgas, 1995; Isen, 2008; Petty & Briñol, 2015). Future studies should consider including a condition with similar thematic content but presented in a more emotionally neutral style to isolate the role of emotional engagement.

Fourth, we found that perceiving the proximal conflict as less unique, believing conflicts can change, and unfreezing of conflict-related beliefs can independently mediate the effect of the intervention on support for conciliatory policies. However, the interplay between these mechanisms merits further investigation. Prior research has found that the relationship between conflict uniqueness perceptions and support for concessions is conditional on people’s underlying beliefs about conflict malleability (Kudish et al., 2015). This suggests that these mechanisms may not operate in isolation but could interact in complex ways to shape attitudes toward conflict resolution. One limitation of our current data is that we did not have baseline measures to explore such effects. Future research could use longitudinal designs or pre- and post-intervention assessments to explore possible causal or interactive relationships between the variables. Additionally, future work could examine how individual differences, such as personality traits (e.g., openness to experience and cognitive flexibility), might moderate the intervention’s impact on these psychological mechanisms.

Fifth, learning about a peace process may have psychological effects beyond those explored in this study. For instance, it could potentially reduce zero-sum perceptions of conflict—the view that one side’s gains necessarily come at the expense of the other side (Maoz & McCauley, 2005). Different aspects of the source case could be strategically emphasized to address this specific belief (and others); for example, how security arrangements in the different conflict benefited both communities. Finally, future research could investigate whether more immersive and prolonged engagement with the different conflict and peace process would produce stronger and more consistent effects. For instance, studies could compare the impact of brief interventions to more intensive experiences, such as a week-long educational trip to Northern Ireland, mirroring real-world initiatives.

## Conclusion

Our findings provide initial evidence that exposure to successful conflict resolution in a different context can influence beliefs about a proximal conflict, though its effectiveness in promoting support for conciliatory policies was inconsistent. Although we found no evidence that learning from resolved conflicts backfire, the variability in impact suggests the need for caution in implementing costly, large-scale intervention programs. Notably, conflict-related beliefs tended to change more among leftists and centrists, compared to rightists. Moreover, our findings revealed that explicit conflict comparisons were largely ineffective, indicating that a more indirect approach—one that initially avoids direct references to the proximal conflict—may be more likely to induce shifts in beliefs and attitudes.

## Notes

1. The final item was not originally part of the uniqueness measure, and was preregistered in Studies 2 and 3 as a one-item “similarity” measure. However, as perceiving the Israeli–Palestinian and Northern Ireland conflicts as similar implies seeing the Israeli–Palestinian conflict as less unique, we included it in this measure. Moreover, the reliability of the uniqueness measure increased across all studies with the similarity item included.

2. Due to null results in Study 1, we included hope as an “additional variable” in our preregistration in Studies 2–4, rather than as a hypothesized mediator. However, given its theoretical relevance, we report hope in our main findings for consistency across studies.

3. We report two-tailed tests (despite preregistering one-tailed tests) for a more conservative and consistent approach.
